# Long-term effectiveness of inpatient and day hospital treatment in departments of psychosomatic medicine and psychotherapy in Germany

**DOI:** 10.3389/fpsyt.2025.1531504

**Published:** 2025-10-29

**Authors:** Henrik Kessler, Stephan Doering, Aram Kehyayan, Magdalena Pape, Tobias Hofmann, Matthias Rose, Katrin Imbierowicz, Franziska Geiser, Ilona Croy, Kerstin Weidner, Jörg Rademacher, Silke Michalek, Eva Morawa, Yesim Erim, Christoph Jansen, Martin Teufel, Stanislav Heinzmann, Claas Lahmann, Eva Milena Johanne Peters, Johannes Kruse, Dirk von Boetticher, Christoph Herrmann-Lingen, Mariel Nöhre, Martina de Zwaan, Ulrike Dinger, Hans-Christoph Friederich, Alexander Niecke, Christian Albus, Rüdiger Zwerenz, Manfred Beutel, Casper Roenneberg, Peter Henningsen, Barbara Stein, Christiane Waller, Karsten Hake, Carsten Spitzer, Andreas Stengel, Stephan Zipfel, Katja Weimer, Harald Gündel, Stephan Herpertz

**Affiliations:** ^1^ Department of Psychosomatic Medicine and Psychotherapy, LWL University Hospital, Ruhr-University Bochum, Bochum, Germany; ^2^ Department of Psychosomatic Medicine and Psychotherapy, Philipps-University of Marburg, Fulda, Germany; ^3^ Department of Psychoanalysis and Psychotherapy, Medical University of Vienna, Vienna, Austria; ^4^ Department of Clinical Psychology and Psychotherapy, University of Bamberg, Bamberg, Germany; ^5^ Charité Center for Internal Medicine and Dermatology, Department of Psychosomatic Medicine, Charité-Universitätsmedizin Berlin, Corporate Member of Freie Universität Berlin and Humboldt-Universität zu Berlin, Berlin, Germany; ^6^ Department of Psychosomatic Medicine and Psychotherapy, DRK Kliniken Berlin Wiegmann Klinik, Berlin, Germany; ^7^ Department of Psychosomatic Medicine and Psychotherapy, University Hospital Bonn, University of Bonn, Bonn, Germany; ^8^ Department of Psychotherapy and Psychosomatic Medicine, Carl Gustav Carus Faculty of Medicine, Technische Universität, Dresden, Germany; ^9^ Department of Clinical Psychology, Friedrich-Schiller University, Jena, Germany; ^10^ Department of Psychosomatic Medicine and Psychotherapy, LVR-University Hospital, Heinrich Heine University Düsseldorf, Düsseldorf, Germany; ^11^ Department of Psychosomatic Medicine and Psychotherapy, University Hospital of Erlangen, Friedrich-Alexander University Erlangen-Nuremberg, Erlangen, Germany; ^12^ Clinic of Psychosomatic Medicine and Psychotherapy, LVR-University Hospital, University of Duisburg-Essen, Essen, Germany; ^13^ Center for Translational Neuro- and Behavioral Sciences, University of Duisburg-Essen, Essen, Germany; ^14^ Department of Psychosomatic Medicine und Psychotherapy, Medical Center - University of Freiburg, Faculty of Medicine, University of Freiburg, Freiburg, Germany; ^15^ Department of Psychosomatic Medicine and Psychotherapy, Justus-Liebig University of Giessen, Giessen, Germany; ^16^ Department of Psychosomatic Medicine and Psychotherapy, Philipps-University of Marburg, Marburg, Germany; ^17^ Department of Psychosomatic Medicine and Psychotherapy, University of Göttingen Medical Centre, Göttingen, Germany; ^18^ Department of Psychosomatic Medicine and Psychotherapy, Hannover Medical School, Hannover, Germany; ^19^ Department of General Internal Medicine and Psychosomatics, University Hospital, Heidelberg University, Heidelberg, Germany; ^20^ Department of Psychosomatic Medicine and Psychotherapy, University of Cologne, Faculty of Medicine and University Hospital Cologne, Cologne, Germany; ^21^ Department of Psychosomatic Medicine and Psychotherapy, University Medical Center of the Johannes Gutenberg University Mainz, Mainz, Germany; ^22^ Department of Psychosomatic Medicine and Psychotherapy, University Hospital, Technical University of Munich, Munich, Germany; ^23^ Department of Psychosomatic Medicine and Psychotherapy, Paracelsus Medical University, Nuremberg General Hospital, Nuremberg, Germany; ^24^ Department of Psychosomatic Medicine and Psychotherapy, University Medical Center Rostock, Rostock, Germany; ^25^ Internal Medicine VI, Psychosomatic Medicine and Psychotherapy, University Hospital Tübingen, Tübingen, Germany; ^26^ German Center for Mental Health, Site Tübingen, Tübingen, Germany; ^27^ Clinic for Psychosomatic Medicine and Psychotherapy, Klinikum Stuttgart, Stuttgart, Germany; ^28^ Department of Psychosomatic Medicine and Psychotherapy, Ulm University Medical Center, Ulm, Germany

**Keywords:** inpatient treatment, psychotherapy, psychosomatic medicine, effectiveness, outcome, follow-up

## Abstract

**Background:**

There is a lack of reliable data concerning the long-term effectiveness of psychosomatic inpatient and day hospital treatment in a naturalistic setting. The Multicenter Effectiveness Study of Inpatient and Day Hospital Treatment in Departments of Psychosomatic Medicine and Psychotherapy in Germany aims to provide such data. The study itself and effectiveness from admission to discharge have already been reported in this journal (Doering et al., 2023). This brief report adds 12-month follow-up data.

**Methods:**

The relevant outcome variables concerning somatoform, trauma-related, eating and personality disorders, as well as anxiety and depressive disorders were assessed by means of questionnaires on admission (T0), at discharge (T1) and after 12 months (T2). In order to make targeted statements about effectiveness regarding only clinically relevant symptoms, each symptom domain was stratified by severity at admission.

**Results:**

From a total of 2,094 patients at admission, 60.6% still provided data at T2. Overall, the changes achieved at discharge (T1) already reported in Doering (2023) remained stable over the 12-month follow-up period (T2). There were hence significant improvements from T0 to T2 across all symptom domains with large effect sizes ranging from d=1.0 to 3.4.

**Conclusions:**

The already reported effectiveness of inpatient and day hospital treatment in German university departments of Psychosomatic Medicine and Psychotherapy in a naturalistic setting is further strengthened by providing evidence for sustained treatment effects over the 12-month follow-up period. Importantly, the entire spectrum of disorders investigated showed this pattern.

**Clinical Trial Registration:**

https://drks.de/search/de/trial/DRKS00016412 RKS00016412, identifier DRKS00016412.

## 1 Introduction

This brief research report mainly adds 12-month follow-up data to the article of Doering et al. (1) published in this journal and describing “The Multicenter Effectiveness Study of Inpatient and Day Hospital Treatment in Departments of Psychosomatic Medicine and Psychotherapy in Germany (MEPP)”. Hence, details about the study itself can be found there.

Inpatient and day hospital psychosomatic-psychotherapeutic treatment in Germany is geared towards patients with complex psychosomatic and somato-psychic disorders, predominantly with long-standing and chronic illness courses. The most common diagnoses include somatoform and dissociative disorders, trauma-related disorders, eating disorders, personality disorders, as well as anxiety and depressive disorders. A high proportion of patients have comorbid somatic diseases. The inpatient or day hospital treatment, lasting typically six to eight weeks, is guideline-oriented, multimodal, and includes psychotherapeutic, psychopharmacological, and, if necessary, somatic interventions. In addition to individual psychotherapy sessions, various group and creative therapies are offered (totaling 15–20 hours of interventions per week). Many different therapy components (e.g., individual psychotherapy, group therapy) have demonstrated their efficacy individually in randomized controlled trials (RCTs) in specific patient populations (2). Evidence for the effectiveness of the above-mentioned multimodal approach as a whole package, however, is sparse in acute care. This approach and the long-term scope of treatment is a particularity of the German system of Psychosomatic Medicine and Psychotherapy. Part of this is grounded in the fact that in many countries longer-term inpatient treatment for psychosomatic patients is not covered by health care insurances.

There are only few findings regarding effectiveness, which include data from acute clinical care settings, primarily based on naturalistic studies (mostly with smaller samples). The meta-analysis by Liebherz and Rabung (3) covering a total of 109 studies of psychotherapeutic hospital treatments reports an average effect size of d=0.4-0.7, but criticizes the mostly poor quality of the individual studies. The study by Valdés-Stauber et al. (4) reports an effect size of d=1.1 for inpatient psychosomatic treatment two years post-discharge; however, this is based on a relatively small sample (N = 250) from only one clinic.

Therefore, the aim of the present multicenter study is to investigate the effectiveness of inpatient and day hospital psychosomatic-psychotherapeutic treatments with a follow-up of one year post-treatment in a larger sample. To this end we collected a representative sample of more than 2000 patients treated in 19 departments of Psychosomatic Medicine and Psychotherapy at German university hospitals. The large sample size allowed for sub-group analyses in patients with symptoms of somatoform disorders, posttraumatic stress disorder (PTSD), eating disorders, personality dysfunction as well as depression and anxiety. Assessments were conducted by means of standardized questionnaires and at three time points: on admission, before discharge, and at follow-up one year after discharge. We chose a naturalistic prospective study design in order (a) to ensure a large sample size including the vast majority of German university departments of Psychosomatic Medicine and Psychotherapy, (b) to obtain ecological validity by evaluating actual mental health provision and avoiding patient selection needed for RCTs and finally (c) to avoid the ethical problem of not treating patients in need due to the necessity of waiting-list controls.

## 2 Method

This study was approved by the ethics committee of the medical faculty of the Ruhr-University Bochum on October 17, 2018 (ID: 18-6388, the approval was confirmed by the ethics committees of all participating universities) and was registered at the German Clinical Trials Register (DRKS, www.drks.de; ID: DRKS00016412).

The Multicenter Effectiveness Study of Inpatient Psychosomatic-Psychotherapeutic Treatments (MEPP Study) presented here assesses the treatment effectiveness at 19 German Psychosomatic University Hospitals under naturalistic conditions, including a 12-month follow-up, with a total of N = 2,094 prospectively enrolled unselected patients. In contrast to the *efficacy* in RCTs, the central measure is the *effectiveness* (i.e. under real-world conditions without patient selection and randomization) of the entire multimodal treatment in this cohort study. Details of the study design and pre-post results of the MEPP Study (before treatment and directly at discharge) were presented in Doering et al. (1, 5). This article adds data from the 12-month follow-up. Due to the diversity of complaints in this heterogeneous sample, a range of psychological symptoms was assessed using six internationally established questionnaires (depression, anxiety, somatization, symptoms of eating disorders and post-traumatic stress disorder, personality function). More specifically, primary outcome criteria were assessed with the German version of the Patient Health Questionnaire (PHQ-D) (6) containing three subscales to assess depression (PHQ-9), anxiety (GAD-7), and somatization (PHQ-15). Additionally, post-traumatic stress disorder was assessed by the German version of the PTSD Checklist for DSM-5 (PCL-5) (7), eating disorder psychopathology with the Eating Disorder Examination – Questionnaire (EDE-Q) (8) and personality functioning by the 12-item short version of the Structure Questionnaire of the Operationalized Psychodynamic Diagnosis (OPD-SQS; German: OPD-SFK) (9). Since not all patients exhibited clinically relevant severity levels in all symptom areas, each symptom area was stratified by severity at admission (**Table 1**) to make targeted statements about effectiveness regarding only clinically relevant symptoms. Importantly, for the sake of differentiation of possible treatment effects, patients were divided by dimensional symptom load (questionnaires) *not* categorical diagnoses (clinical interviews). Consequently, some patients with e.g. categorical eating disorders might have had only mild depression symptoms (questionnaire) at admission and vice versa. The questionnaires were administered at admission (T0), discharge (T1, on average after 53.8 [SD 23.0] days for full inpatient and 46.5 [SD 20.2] days for day hospital treatment, including treatment-free weekends), and one year after discharge (T2). Intention-to-treat analyses were conducted across all N = 2,094 patients. Missing data were replaced by imputation (Markov Chain Monte Carlo method with predictive mean matching, 20 imputations). Results without imputation will also be mentioned briefly.

**Table 1:** Mean changes between admission (T0), discharge (T1), and 12-month follow-up (T2).

	N (%)	T0 Mean (SD)	T1 Mean (SD)	T2 Mean (SD)	t	df	p	d
Depression (PHQ-9), Total Score								
None (<5)	99 (4.7%)	2.78 (1.37)	3.92 (3.49)	5.21 (5.22)	-4.476	98	<0.001	-1.25
Mild (5-9)	331 (15.9%)	7.29 (1.33)	6.15 (3.51)	7.13 (4.71)	0.615	330	0.539	0.09
Moderate (10-14)	527 (25.3%)	12.04 (1.37)	8.33 (4.16)	9.43 (5.47)	10.847	526	<0.001	1.42
Moderate-Severe (15-19)	627 (30.1%)	16.99 (1.39)	11.23 (5.11)	11.89 (6.07)	21.100	626	<0.001	2.79
Severe (≥20)	499 (23.9%)	22.32 (1.94)	14.93 (5.79)	15.44 (6.36)	24.999	498	<0.001	2.91
Anxiety (GAD-7), Total Score								
None (<5)	181 (8.7%)	2.58 (1.29)	3.35 (2.66)	4.09 (3.97)	-4.947	180	<0.001	-0.85
Mild (5-9)	553 (26.5%)	7.21 (1.38)	5.72 (3.50)	6.07 (4.13)	6.458	552	<0.001	0.64
Moderate (10-14)	694 (33.3%)	12.01 (1.44)	8.11 (4.26)	8.50 (4.78)	19.272	693	<0.001	1.86
Severe (15-21)	656 (31.5%)	17.42 (1.90)	10.87 (5.22)	11.64 (5.46)	27.693	655	<0.001	2.46
Somatization (PHQ-15), Total Score								
None (<5)	115 (5.5%)	3.21 (1.11)	4.05 (3.09)	5.56 (4.64)	-5.291	114	<0.001	-1.51
Mild (5-9)	460 (22.1%)	7.29 (1.35)	6.92 (3.31)	7.74 (4.50)	-2.204	459	0.028	-0.27
Moderate (10-14)	664 (31.8%)	12.02 (1.36)	9.98 (4.04)	10.24 (4.79)	9.897	663	<0.001	1.06
Severe (15-30)	830 (39.8%)	18.70 (3.04)	15.11 (4.92)	15.00 (5.44)	20.253	829	<0.001	1.06
Eating Disorder (EDE-Q), Total Mean								
None (<1)	824 (39.5%)	0.32 (0.29)	0.35 (0.47)	0.63 (0.88)	-10.452	823	<0.001	-0.87
Mild (1-1.99)	415 (19.9%)	1.42 (0.30)	1.24 (0.91)	1.35 (1.11)	1.236	414	0.217	0.19
Moderate (2-2.99)	300 (14.4%)	2.44 (0.28)	2.16 (1.01)	2.21 (1.21)	3.326	299	0.001	0.64
Moderate-Severe (3-3.99)	283 (13.6%)	3.43 (0.28)	2.66 (1.06)	2.79 (1.31)	8.330	282	<0.001	1.80
Severe (≥4)	263 (12.6%)	4.82 (0.57)	3.81 (1.15)	3.83 (1.32)	12.083	262	<0.001	1.36
PTSD Symptoms (PCL-5), Total Score								
None (<30)	961 (46.1%)	15.01 (9.24)	14.43 (12.89)	15.71 (14.80)	-1.420	960	0.156	-0.06
Mild (30-39)	351 (16.8%)	34.55 (2.82)	25.62 (14.79)	25.69 (16.76)	10.067	350	<0.001	2.45
Moderate (40-49)	292 (14.0%)	44.35 (2.81)	34.30 (15.83)	33.29 (17.06)	11.145	291	<0.001	2.97
Moderate-Severe (50-59)	264 (12.7%)	54.01 (2.94)	39.63 (17.25)	39.68 (17.87)	12.798	263	<0.001	3.40
Severe (≥60)	214 (10.3%)	66.82 (5.52)	52.22 (16.07)	50.12 (18.39)	13.053	213	<0.001	2.24
Personality Structure Pathology (OPD-SFK), Total Score								
None (≤10)	169 (8.1%)	6.49 (2.95)	7.62 (6.02)	8.85 (6.39)	-4.733	168	<0.001	-0.63
Mild (11-20)	498 (23.9%)	16.15 (2.76)	14.85 (7.06)	14.78 (7.92)	3.853	497	<0.001	0.39
Moderate (21-30)	781 (37.5%)	25.70 (2.82)	22.59 (7.86)	22.34 (9.00)	10.691	780	<0.001	0.96
Severe (≥31)	635 (30.5%)	36.66 (4.38)	30.93 (8.40)	30.89 (8.88)	17.662	634	<0.001	1.19

Outcome Measures. Mean changes between admission (T0), discharge (T1), and 12-month follow-up (T2). N = 2094. Paired samples t-test between T0 and T2 (two-tailed). Effect sizes for repeated measures (according to Morris & DeShon, 2002). Questionnaires used: Depression PHQ-9 (Patient Health Questionnaire), Anxiety GAD-7 (Generalized Anxiety Disorder Scale), Somatization PHQ-15, Eating Disorder EDE-Q (Eating Disorder Examination Questionnaire), Posttraumatic Stress Disorder PCL-5 (PTSD Checklist for DSM-5), Personality Structure Pathology OPD-SFK (Operationalized Psychodynamic Diagnosis – Structure Questionnaire Short Version).

## 3 Results

**Figure 1:**
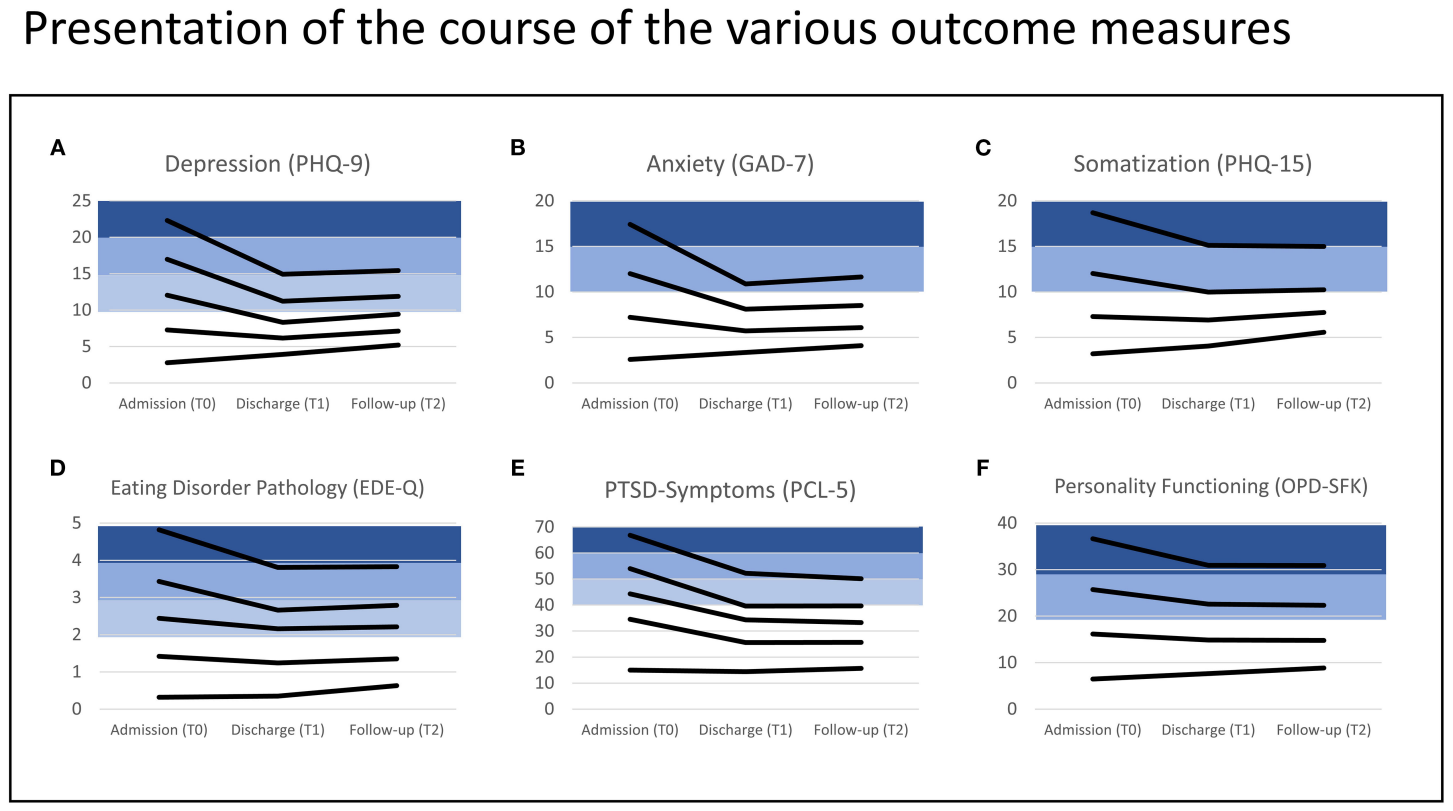
Representation of the course of the various outcome measures (**A**: PHQ-9; **B**: GAD-7; **C**: PHQ-15; **D**: EDE-Q; **E**: PCL-5; **F**: OPD-SFK) over three time points (T0, T1, T2). Each line represents the trajectory of mean scores for the respective severity level (corresponding to data in **Table 1**). The lower strata in white represent the range with no or low symptom severity. Above the threshold for clinical relevance, the strata are colored in shades of blue.

Details about the patient sample at baseline have been published in (5). In our previous article in this journal (1), we provided detailed information about patient flow, characteristics, diagnoses and variables of treatment. Focusing here on the 12-month follow-up, 60.6% of the original patient sample at admission were still captured for data analysis. **Table 1** displays the changes in various symptom domains from admission (T0), through discharge (T1), to the 12-month follow-up (T2), stratified by severity at admission. Overall, a relatively homogeneous pattern was observed across the different outcome measures: Beginning from a moderate to severe pathology at admission, there was a consistent clinically and statistically significant improvement in symptoms. The significant improvement from T0 to T1 has already been reported in (1). The significant improvement from T0 to T2 is displayed in **Table 1** with large effect sizes ranging from d=1.0 to 3.4 (with one exception in the case of eating disorder symptoms). Importantly, differences between T1 and T2 in relevant symptom severity levels were mostly not significant with the exception of slight but significant differences in moderate to severe depression and moderate to severe anxiety, where the means increased at T2 (**Table 1**). Overall, this indicates that the changes achieved at discharge (T1) remained stable over the 12-month follow-up period (T2). In addition to the reported effect sizes per stratum, **Table 1** also illustrates that after treatment, patients often transitioned to the next lower stratum, indicating a clinically relevant response from a categorical perspective as well. **Figure 1** is a graphical version of the data provided in **Table 1** in order to better depict the change in means over time. **Figures 1A–F** represent the different symptom domains and each line displays a specific severity level. In depression pathology as measured by PHQ-9, for instance, there are five severity levels (from none <5 to severe ≥20), see **Table 1**, which are represented by five lines in **Figure 1A**). The PHQ-9 mean scores in the three columns of **Table 1** (T0, T1, T2) are displayed as three data points within the line of the respective severity level.

We additionally checked the effect of imputation by calculating all data reported in **Table 1** a second time with the original data set (no imputation, missing data). The results across all symptom domains and severity levels were statistically comparable to the data reported in **Table 1**, and are reported in **Supplementary Table 1**.

We also performed a comparison between patients who participated in the follow-up and those who did not. There was no difference in gender distribution, but those who did not participate in the follow-up were younger, and showed higher symptom severity at baseline (T0) for somatization, anxiety, and PTSD symptoms, but not for depression, eating disorder pathology, or personality functioning.

Concerning pre-/post treatment effects, no differences were found between patients who participated in the follow-up and those who did not regarding depression, anxiety, somatization, and eating disorder pathology. Repeated measures ANOVA showed a significant between-subjects effect for personality functioning (lower personality functioning in the group that did not participate in the follow-up), as well as a between-subjects effect for PTSD symptoms (higher symptom load in the group that did not participate in the follow-up). Importantly, there were no interaction effects (Time*Group) in any of the measures, implying that the time course of symptom severity from T0 to T1 did not differ between the groups. These additional analyses are now reported in the **Supplementary Materials, Tables 2–4**.

## 4 Discussion

The MEPP study is the largest prospective and naturalistic multicenter project investigating inpatient and day hospital psychosomatic-psychotherapeutic treatments in the majority of German university departments of Psychosomatic Medicine and Psychotherapy. In addition to the significant and clinically relevant treatment effects directly at discharge reported in our previous paper in *Frontiers in Psychiatry* (1), this article strengthens the claimed effectiveness of multimodal psychosomatic-psychotherapeutic treatment by providing evidence for sustained treatment effects over the 12-month follow-up period. The study also shows that the entire spectrum of disorders investigated could be affected by treatment, with the strongest effects observed in depression, anxiety, and PTSD symptomatology, and comparatively lower effects in somatization, eating disorder pathology, and personality structure (see table of effect sizes). Changes in the non-pathological range can be partially explained by “regression to the mean”. Even considering the usual spontaneous remissions of untreated patients with effect sizes of 0.1-0.15 (10, 11), a significant effect of the treatment can still be assumed. By stratifying the unselected sample by symptomatology (e.g. depression, somatization) and severity (e.g. moderate, severe), the results can be compared with those of selected samples in the context of RCTs. This approach explains the significantly higher effect sizes than those reported in the meta-analysis by Liebherz and Rabung (3) (d=0.4-0.7) considering only non-stratified samples. In terms of effect sizes, our study is comparable to the study by Valdés-Stauber et al. (4) (naturalistic, prospective, follow-up). We were able to replicate their good results and stability in follow-up in our multicenter study with a significantly larger sample size, thereby confirming the effectiveness of psychosomatic-psychotherapeutic treatment under real-world conditions.

Discussion of the possible meaning of treatment effects across various symptom areas, somatization, depression, PTSD, and others, is provided in (1). Considering limitations, the lack of a control group, and the inclusion of only a part of the initial sample for the follow-up (60,6%), should be mentioned. As for the control group, ethical considerations (number of untreated patients in waiting-list necessary) have influenced our decision. Considering the drop-out rate, our result is within the range reported in comparable naturalistic 12-month follow-up studies of treatment effects [e.g. Valdes-Stauber et al. with 45% (4); Nübling et al. with 62% (12)]. In our view, our approach with imputation of missing data adequately mended the data gap, and is consistent with the data reported in our previous publications regarding this multicenter study (1, 5). Additionally, patients who participated in the follow-up were compared to those who did not. It can be stated that treatment effects were similar between follow-up completers and non-completers, but non-completers were younger and had higher symptom load in regard to somatization, anxiety, and PTSD symptoms.

Overall, the follow-up data presented here support previous findings and show a sustained effect of psychosomatic-psychotherapeutic inpatient- and day-hospital-treatment over a period of 12 months. Further analyses of the comprehensive data sample acquired through the MEPP study are in preparation, and will include analyses of biological parameters, as well as more detailed looks into specific patient subgroups.

## Data Availability

The datasets presented in this article are not readily available because the European General Data Protection Regulation (GDPR) does not allow to share personal data of patients publicly (https://gdpr.eu). The ethics commissions of all of the study centers have approved the study under the condition that even the transfer of data from the German sites to the Austrian PI (SD) can only take place according to specific security regulations. Requests to access these datasets should be directed to Stephan Doering, stephan.doering@meduniwien.ac.
