# Supplementary material for: Long-term effectiveness of inpatient and day hospital treatment in departments of psychosomatic medicine and psychotherapy in Germany
Source: Front Psychiatry. 2025 Oct 29;16:1531504. doi: 10.3389/fpsyt.2025.1531504 (PMC12605911; doi:10.3389/fpsyt.2025.1531504)
Supplement: Supplementary file 1 [file Table1.docx]

Supplementary Table 1: Outcome Measures. Mean changes between admission (T0), discharge (T1), and 12-month follow-up (T2). N=1270, without imputation of missing data. Paired samples t-test between T0 and T2 (two-tailed). Effect sizes for repeated measures (according to Morris & DeShon, 2002). Questionnaires used: Depression PHQ-9 (Patient Health Questionnaire), Anxiety GAD-7 (Generalized Anxiety Disorder Scale), Somatization PHQ-15, Eating Disorder EDE-Q (Eating Disorder Examination Questionnaire), Posttraumatic Stress Disorder PCL-5 (PTSD Checklist for DSM-5), Personality Structure Pathology OPD-SFK (Operationalized Psychodynamic Diagnosis – Structure Questionnaire Short Version).

|  | **n (%)** | **T0 Mean (SD)** | **T1 Mean (SD)** | **T2 Mean (SD)** | **t** | **df** | **p** | **d** |
| --- | --- | --- | --- | --- | --- | --- | --- | --- |
|  |  |  |  |  |  |  |  |  |
| Depression (PHQ-9), Total Score |  |  |  |  |  |  |  |  |
| None (<5) | 57  (4.5%) | 2.80  (1.38) | 3.58  (3.66) | 5.09  (5.97) | -2.784 | 56 | 0.007 | -0.98 |
| Mild (5-9) | 212  (16.7%) | 7.29  (1.36) | 6.23  (3.59) | 7.48  (4.69) | -0.588 | 211 | 0.557 | 0.27 |
| Moderate (10-14) | 323  (25.4%) | 12.02  (1.39) | 8.47  (4.26) | 9.69  (5.32) | 7.724 | 322 | <0.001 | 1.75 |
| Moderate-Severe (15-19) | 363  (28.6%) | 16.95  (1.40) | 10.86  (5.03) | 11.65  (5.91) | 17.007 | 362 | <0.001 | 2.82 |
| Severe (≥20) | 294  (23.1%) | 22.30  (1.96) | 15.06  (5.92) | 15.90  (6.47) | 17.445 | 293 | <0.001 | 2.65 |
|  |  |  |  |  |  |  |  |  |
| Anxiety (GAD-7), Total Score |  |  |  |  |  |  |  |  |
| None (<5) | 115  (9.1%) | 2.58  (1.33) | 3.36  (2.79) | 4.14  (4.22) | -3.854 | 114 | <0.001 | -0.86 |
| Mild (5-9) | 355  (28.0%) | 7.23  (1.37) | 5.75  (3.49) | 6.40  (4.25) | 3.675 | 354 | <0.001 | 0.47 |
| Moderate (10-14) | 403  (31.7%) | 12.07  (1.41) | 8.11  (4.17) | 8.53  (4.70) | 15.083 | 402 | <0.001 | 1.92 |
| Severe (15-21) | 374  (29.4%) | 17.34  (1.89) | 10.90  (5.19) | 11.68  (5.61) | 19.843 | 372 | <0.001 | 2.39 |
|  |  |  |  |  |  |  |  |  |
| Somatization (PHQ-15), Total Score |  |  |  |  |  |  |  |  |
| None (<5) | 70  (5.5%) | 3.12  (1.23) | 4.01  (3.25) | 5.16  (4.97) | -3.297 | 69 | 0.002 | -1.15 |
| Mild (5-9) | 297  (23.4%) | 7.37  (1.37) | 7.06  (3.36) | 7.84  (4.47) | -1.840 | 296 | 0.067 | 0.27 |
| Moderate (10-14) | 406  (32.0%) | 11.97  (1.33) | 9.89  (3.87) | 10.36  (4.69) | 7.129 | 405 | <0.001 | 0.98 |
| Severe (15-30) | 477  (37,6%) | 18.59  (2.96) | 15.06  (4.91) | 15.17  (5.47) | 14.383 | 476 | <0.001 | 1.02 |
|  |  |  |  |  |  |  |  |  |
| Eating Disorder (EDE-Q), Total Mean |  |  |  |  |  |  |  |  |
| None (<1) | 484  (38.1%) | 0.33  (0.29) | 0.34  (0.45) | 0.62  (0.92) | -7.138 | 483 | <0.001 | -0.83 |
| Mild (1-1.99) | 247  (19.4%) | 1.43  (0.30) | 1.23  (0.82) | 1.39  (1.05) | 0.653 | 246 | 0.514 | 0.11 |
| Moderate (2-2.99) | 179  (14.1%) | 2.43  (0.28) | 2.09  (1.05) | 2.26  (1.19) | 1,865 | 178 | 0.051 | 0.49 |
| Moderate-Severe (3-3.99) | 170  (13.4%) | 3.44  (0.28) | 2.71  (0.99) | 2.80  (1.27) | 6.709 | 167 | <0.001 | 1.85 |
| Severe (≥4) | 150  (11.8%) | 4.82  (0.60) | 3.75  (1.22) | 3.76  (1.37) | 9.011 | 149 | <0.001 | 1.43 |
|  |  |  |  |  |  |  |  |  |
| PTSD Symptoms (PCL-5), Total Score |  |  |  |  |  |  |  |  |
| None (<30) | 575  (45.3%) | 15.27  (9.17) | 15.25  (13.48) | 16.42  (15.25) | -1.277 | 538 | 0.202 | -0.10 |
| Mild (30-39) | 202  (15.9%) | 34.54  (2.78) | 26.41  (14.67) | 26.45  (15.37) | 7.416 | 190 | <0.001 | 2.29 |
| Moderate (40-49) | 161  (12.7%) | 44.27  (2.81) | 35.56  (14.73) | 32.95  (16.21) | 8.862 | 153 | <0.001 | 3.27 |
| Moderate-Severe (50-59) | 145  (11.4%) | 53.71  (3.04) | 40.95  (16.04) | 40.19  (18.21) | 8.581 | 142 | <0.001 | 2.93 |
| Severe (≥60) | 111  (8.7%) | 66.56  (5.37) | 53.43  (15.83) | 51.22  (17.93) | 9.228 | 110 | <0.001 | 2.30 |
|  |  |  |  |  |  |  |  |  |
| Personality Structure Pathology (OPD-SFK), Total Score |  |  |  |  |  |  |  |  |
| None (≤10) | 110  (8.7%) | 6.41  (2.82) | 7.12  (5.43) | 8.76  (6.48) | -3.353 | 99 | 0.001 | -0.68 |
| Mild (11-20) | 300  (23.6%) | 16.16  (2.83) | 14.71  (7.06) | 15.31  (7.92) | 1.636 | 281 | 0.103 | 0.23 |
| Moderate (21-30) | 482  (38.0%) | 25.67  (2.81) | 22.73  (7.80) | 22.70  (8.80) | 7.342 | 448 | <0.001 | 0.82 |
| Severe (≥31) | 360  (28.3%) | 36.71  (4.50) | 30.92  (8.45) | 30.90  (8.95) | 12.741 | 337 | <0.001 | 1.16 |

Supplementary Table 2: Comparison of Gender Distribution between patients who participated in the follow-up (“Completers”) and those who did not (“Non-completers”). Cross Tables with Chi-Squared test.

| **Gender** | **Non-Completers** | **Completers** | **Total** |
| --- | --- | --- | --- |
|  |  |  |  |
| Female | 542 | 882 | 1424 |
| Male | 272 | 338 | 660 |
| Other | 1 | 0 | 1 |
| Total | 815 | 1270 | 2085 |
|  |  |  |  |
| **Pearson Chi Squared** | **df** | **p** |  |
| 3.439 | 2 | 0.179 |  |

Supplementary Table 3: Differences between Completers and Non-completers, regarding age and baseline symptom severity (T0); means and two-sample t-test

|  | **Non-Completers (SD)** | **n** | **Completers (SD)** | **n** | **T** | **df** | **p** |
| --- | --- | --- | --- | --- | --- | --- | --- |
| Age | 39.13 (14.06) | 815 | 40.38 (14.28) | 1268 | -1.967 | 2081 | 0.049 |
| Depression (PHQ-9), T0 | 14.99 (5.95) | 785 | 14.65 (5.92) | 1249 | 1.249 | 2032 | 0.212 |
| Anxiety (GAD-7), T0 | 11.89 (5.05) | 785 | 11.40 (5.03) | 1247 | 2.167 | 2030 | 0.030 |
| Somatization (PHQ-15), T0 | 13.41 (5.62) | 786 | 12.91 (5.45) | 1250 | 2.023 | 2034 | 0.043 |
| Eating Disorder (EDE-Q), T0 | 1.87 (1.63) | 776 | 1.83 (1.59) | 1230 | 0.483 | 2004 | 0.629 |
| PTSD (PCL-5), T0 | 34.56 (20.17) | 724 | 31.88 (19.27) | 1194 | 2.903 | 1916 | 0.004 |
| Personality Structure (OPD-SFK), T0 | 25.70 (9.86) | 784 | 24.87 (10.01) | 1252 | 1.833 | 2034 | 0.067 |

Supplementary Table 4: Comparison of treatment effects (pre-/post-treatment T0->T1) between Completers and Non-Completers; repeated measures ANOVA

|  | **Effect** | **F** | **p** |
| --- | --- | --- | --- |
| Depression (PHQ-9) | Time | 1141.39 | <0.001 |
|  | Group | 1.371 | 0.242 |
|  | Interaction Time*Group | 0.035 | 0.852 |
|  |  |  |  |
| Anxiety (GAD-7) | Time | 897.11 | <0.001 |
|  | Group | 1.908 | 0.167 |
|  | Interaction Time*Group | 0.809 | 0.368 |
|  |  |  |  |
| Somatization (PHQ-15) | Time | 398.52 | <0.001 |
|  | Group | 0.493 | 0.483 |
|  | Interaction Time*Group | 0.465 | 0.495 |
|  |  |  |  |
| Eating Disorder (EDE-Q) | Time | 149.46 | <0.001 |
|  | Group | 0.234 | 0.629 |
|  | Interaction Time*Group | 1.540 | 0.215 |
|  |  |  |  |
| PTSD (PCL-5) | Time | 219.09 | <0.001 |
|  | Group | 4.364 | 0.037 |
|  | Interaction Time*Group | 3.426 | 0.064 |
|  |  |  |  |
| Personality Structure (OPD-SFK) | Time | 263.70 | <0.001 |
|  | Group | 4.452 | 0.035 |
|  | Interaction Time*Group | 0.493 | 0.483 |
